# Effect of changes in motor skill induced by educational video program to decrease lower-limb joint load during cutting maneuvers: based on musculoskeletal modeling

**DOI:** 10.1186/s12891-024-07642-4

**Published:** 2024-07-09

**Authors:** Sungmin Kim, Jiho So, Youngju Jeon, Jeheon Moon

**Affiliations:** 1 Institute of School Physical Education, Korea National University of Education, Cheongju, Republic of Korea; 2 Digital Health Research Division, Korea Institute of Oriental Medicine, Daejeon, Republic of Korea; 3 Department of Physical Education, Korea National University of Education, Cheongju, Republic of Korea

**Keywords:** Cutting maneuver, Educational video program, Musculoskeletal modeling, Lower extremity joint, Injury prevention

## Abstract

**Background:**

This study investigated the effects of changes in motor skills from an educational video program on the kinematic and kinetic variables of the lower extremity joints and knee ligament load.

**Methods:**

Twenty male participants (age: 22.2 ± 2.60 y; height: 1.70 ± 6.2 m; weight: 65.4 ± 7.01 kg; BMI: 23.32 ± 2.49 $kg/{m}^{2}$) were instructed to run at 4.5 ± 0.2 m/s from a 5 m distance posterior to the force plate, land their foot on the force plate, and perform the cutting maneuver on the left. The educational video program for cutting maneuvers consisted of preparatory posture, foot landing orientation, gaze and trunk directions, soft landing, and eversion angle. The measured variables were the angle, angular velocity of lower extremity joints, ground reaction force (GRF), moment, and anterior cruciate ligament (ACL) and medial collateral ligament (MCL) forces through musculoskeletal modeling.

**Results:**

After the video feedback, the hip joint angles increased in flexion, abduction, and external rotation (*p* < 0.05), and the angular velocity increased in extension (*p* < 0.05). The ankle joint angles increased in dorsiflexion (*p* < 0.05), and the angular velocity decreased in dorsiflexion (*p* < 0.05) but increased in abduction (*p* < 0.05). The GRF increased in the anterior-posterior and medial-lateral directions and decreased vertically (*p* < 0.05). The hip joint moments decreased in extension and external rotation (*p* < 0.05) but increased in adduction (*p* < 0.05). The knee joint moments were decreased in extension, adduction, and external rotation (*p* < 0.05). The abduction moment of the ankle joint decreased (*p* < 0.001). There were differences in the support zone corresponding to 64‒87% of the hip frontal moment (*p* < 0.001) and 32‒100% of the hip horizontal moment (*p* < 0.001) and differences corresponding to 32‒100% of the knee frontal moment and 21‒100% of the knee horizontal moment (*p* < 0.001). The GRF varied in the support zone at 44‒95% in the medial-lateral direction and at 17‒43% and 73‒100% in the vertical direction (*p* < 0.001).

**Conclusions:**

Injury prevention feedback reduced the load on the lower extremity joints during cutting maneuvers, which reduced the knee ligament load, mainly on the MCL.

## Background

To avoid collisions with other players during a sports event, such as soccer, basketball, or rugby, players quickly change the direction of their bodies [1]. This behavior often results in unpredictable situations and is associated with the physical fitness factors of quick response and agility [2]. Cutting maneuvers involve acceleration and deceleration of the body as an ongoing movement controlled and shifted in a new direction [1, 3]. However, if cutting maneuvers are performed repeatedly, the load on the lower extremity joints increases, which can lead to injuries [4]. More than two-thirds of injuries related to cutting maneuvers occur via non-contact mechanisms [5–7]; these cause the most damage, mainly to knee joint ligaments [8]. In a study analyzing 17,397 individuals with musculoskeletal injuries during sports activities in the past decade, 37% (*n* = 6,434) of all patients had knee joint injuries, with the highest proportion (~ 20.3%) attributed to the anterior cruciate ligament (ACL) [9, 10].

The ligaments of the knee joint comprise the ACL, posterior cruciate ligament (PCL), medial collateral ligament (MCL), and lateral collateral ligament (LCL). Their function is to prevent excessive translation and rotation of the knee joint, thereby providing stability to the knee joint and allowing it to perform flexion, extension, and rotation [3, 11]. However, rotational movements, such as cutting maneuvers with a single foot supporting the body weight, require excess force and increase the risk of ACL and MCL injuries [12]. An ACL injury may occur when an individual running at high speeds fails to prevent the tibia from moving forward relative to the femur, resulting in a high impact that increases the load on the knee ligaments [13, 14]. Additionally, because the MCL is relatively less durable than the ACL, PCL, and LCL, an ACL injury can also damage the MCL. Moreover, approximately one-third of these injuries accompany damage to the medial meniscus [15, 16].

To date, several studies on lower extremity injuries have applied muscle strength training programs to strengthen the muscles around the knee joint to effectively prevent lower extremity injuries [17–20]. Although such training may have long-term effects, it is time-consuming and effort-intensive, and its effectiveness may be reduced if the individual is not familiar with the appropriate way to perform the cutting maneuvers [21]. Therefore, it is necessary to find ways to provide athletes with accurate postural feedback to improve their technical aspects for performing cutting maneuvers correctly [21–23].

In previous studies, verbal and auditory feedback have commonly been used to gain short-term effects [23–26]. However, verbal and auditory feedback did not significantly improve the technical aspects and long-term effects [27]. To resolve these limitations, video feedback has been proposed [24, 25]. Video-assisted training can generate interest and motivation among participants, who can learn without temporal or spatial constraints while considering individual levels and learning speeds [25]. Another advantage is that providing verbal, visual, and auditory feedback simultaneously encourages the participants to reproduce accurate movements, thereby maximizing the learning effects [21, 23, 25]. Thus, the effectiveness of training using video feedback may exceed that of other feedback methods, thereby increasing the chances of immediate changes in motor skills.

In this study, we investigated whether video feedback can reduce the load on the lower limb joints in the short term. In the long term, this research can significantly help improve athletes’ performance by preventing knee joint injuries and aiding in achieving their goals [28]. Thus, the study aimed to analyze lower-extremity joint loading and knee ligaments by technical changes from an educational video program during cutting maneuvers using musculoskeletal modeling. The hypotheses of this study were as follows: (1) knee joint angles and angular velocities will increase after video feedback; (2) ground reaction force (GRF) and moment will decrease; (3) the loads on the ACL and MCL will decrease.

## Methods

### Participants

Participants in this study were 20 healthy males (age: 22.2 ± 2.60 years; height: 1.70 ± 6.2 m; weight: 65.4 ± 7.01 kg; BMI: 23.32 ± 2.49 $kg/{m}^{2}$) [29, 30]. The sample size was calculated using the t-test model of G*Power 3.1 based on our pilot study comprising 4 participants [7]. For inclusion in the study, participants needed to have no history of lower extremity injuries and no knee injuries in the preceding six months. The participants were instructed not to engage in strenuous physical activities for 24 h before the experiment. On the day of the experiment, they were given detailed explanations of the experimental procedures and the purpose of the study; those who voluntarily signed an informed consent form participated in the experiment. All participant data were anonymized for privacy protection. The experimental procedure was approved by the Ethics Committee of the Korea National University of Education, and the study protocol was performed in accordance with the Declaration of Helsinki. Written informed consent was obtained from each participant before the start of the experiment.

### Equipment

In this study, seven infrared cameras (Oqus 7+; Qualisys, SWE) were used for a 3D motion analysis, and one force plate (Type 9260AA6; Kistler, SWI) was used to collect kinetic data. The infrared cameras were calibrated to form a global coordinate system, with sampling rates of 150 frames/s for the camera and 1,500 Hz for the ground reaction force (GRF). Two timing gaits (Witty, Microgate, Italy) were installed to measure the speed at each zone.

### Procedures

After arriving at the laboratory, all the participants wore identical lab coats and shoes and reflective markers (1.2 cm in diameter) attached to the main joints and segments of the body in the upper and lower extremities, which was performed according to a previous study [31]. A total of 72 markers were attached, including joint markers for the shoulder, elbow, wrist, knee, and ankle joints; three to four tracking markers for the head, torso, upper arm, lower arm, thigh, calf, and feet; and five markers for the pelvis.

**Fig. 1:**
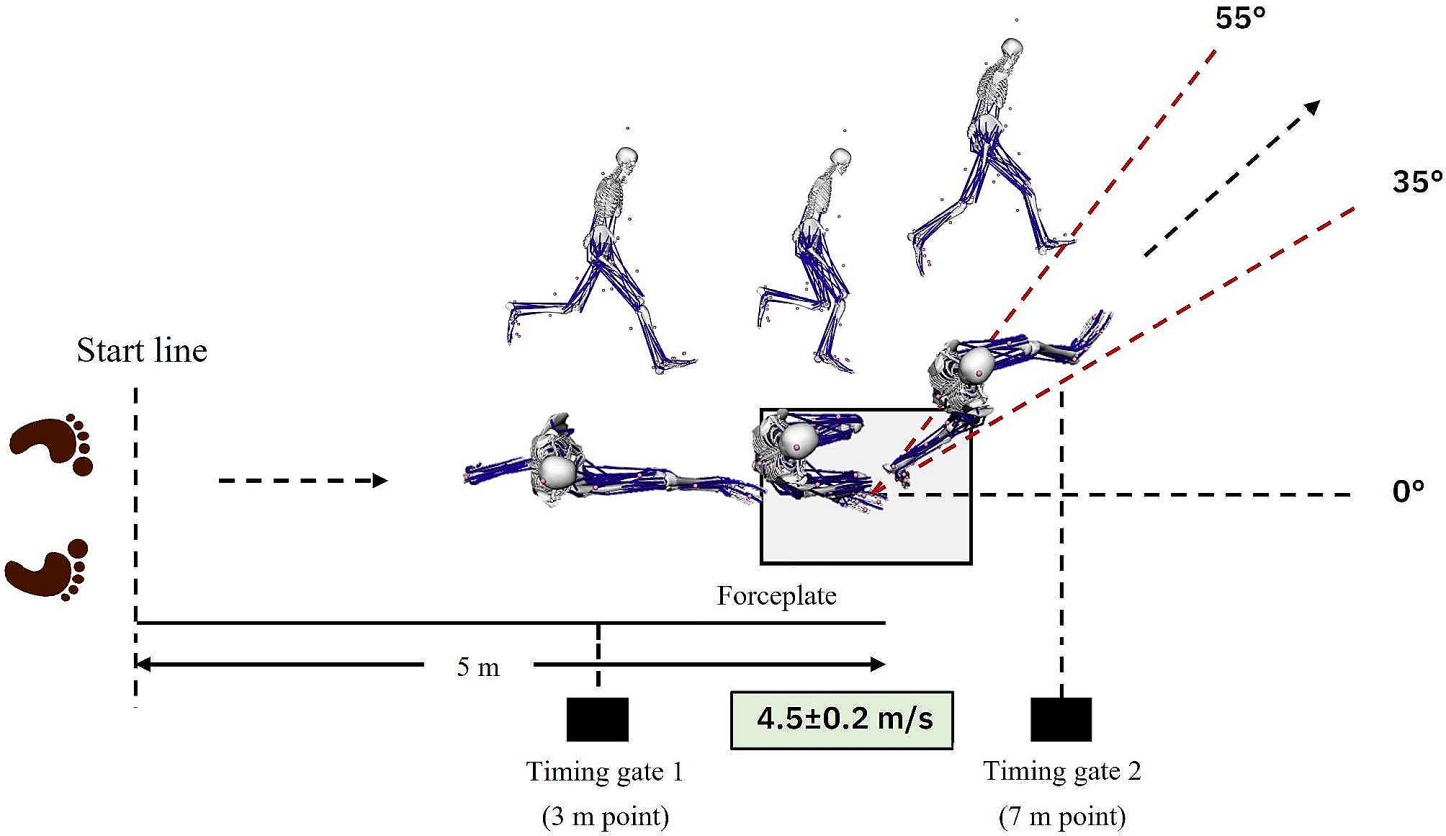
Cutting maneuver task

When the participants signaled that they were ready after sufficient warm-up, the experiment proceeded. To perform the cutting maneuver task, the participants were asked to run at a speed of 4.5 ± 0.2 m/s, starting 5 m behind the force plate. Timing gaits were set up at one zone to control the speed at set levels. The first set-up point was 2 m (from the start line to 3 m points) into the 5 m straight course before the cutting task, and the second one was 2 m (from the start line to 7 m point) after cutting to 5 m at the force plate [32]. The speed measurement began when the participants passed the first timing gate point, and the measurement ended when they passed the second point. A successful performance was defined as running within the set speed range (4.3 ~ 4.7 m/s) for one zone. Also, as the right foot landed on the force plate, only the data of correct movements at 35‒55° to the left of the running direction were collected five times (Fig. 1) [33–35]. We did not control the steps when the participants ran in a straight line, and considered failure if they were out of the range of 35–55° after cutting maneuver or did not run within the specified speed.

### Video feedback

**Fig. 2:**
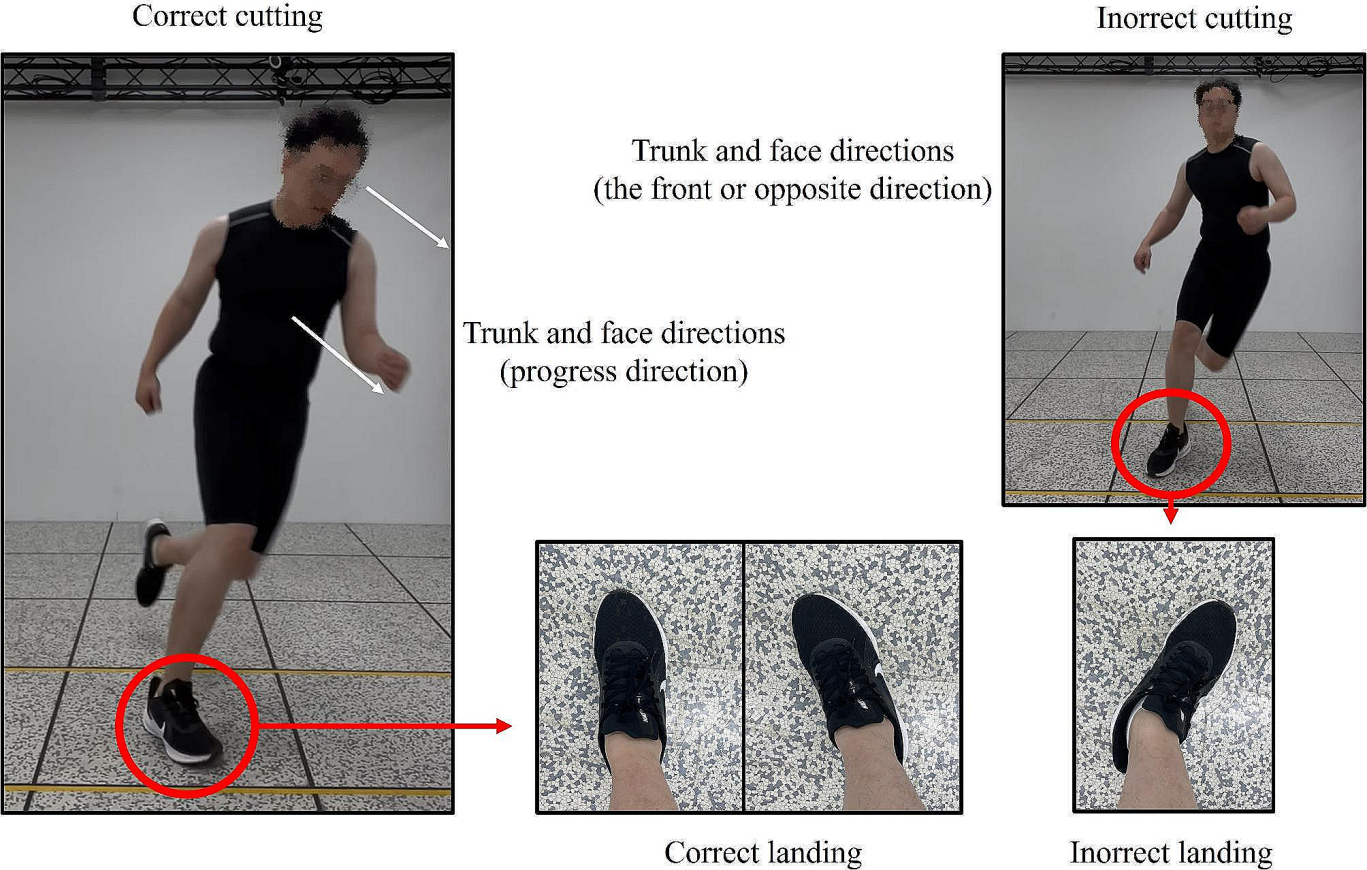
An illustration of video program: Correct posture - the foot in contact with the ground during a turn is oriented in the direction; Incorrect posture - the foot in contact with the ground during a turn is oriented

The injury prevention video program contents for the cutting maneuver in this study were designed based on the results of numerous previous studies. The duration of the video is approximately 5 min, including the mechanisms of athletic injuries and comparing correct and incorrect cutting maneuvers. For ease of understanding, verbal, visual, and auditory feedback were provided simultaneously. The video feedback was designed to ensure that the ankle plantarflexion was directed forward or in the direction of progression during the cutting maneuver and that the gaze and trunk directions were aligned with the ankle plantarflexion [36]. The video feedback had additional content to reduce the force and level of impact on the knee joint through the vertical position of the center of gravity and soft landing motion [37]. It also included the prevention of damage to the ACL, PCL, MCL, LCL, and meniscus by avoiding adduction or abduction of the knee joint (Table 1; Fig. 2) [2, 6, 32, 38, 39].

**Table 1:** Video program contents for cutting maneuver

Index	Time (min)	Video content
Introduction	2	Cases and mechanisms of lower extremity injuries in sports events
Lower extremity athletic injuries: Case analysis	0.5	Cases and mechanisms of injuries related to cutting maneuvers
Description of the cutting maneuver task	1.5	1. Getting ready
		2. The landing foot is directed forward or in the direction of progression [6, 37]
		3. The gaze and trunk directions are aligned with the direction of landing [37, 38]
		4. The knees are bent upon landing (soft landing) [2, 38]
		5. Care should be taken not to increase the knee abduction angle [37, 39]
Wrap-up	1	Review of the entire video with a checklist

### Data analysis

**Fig. 3:**
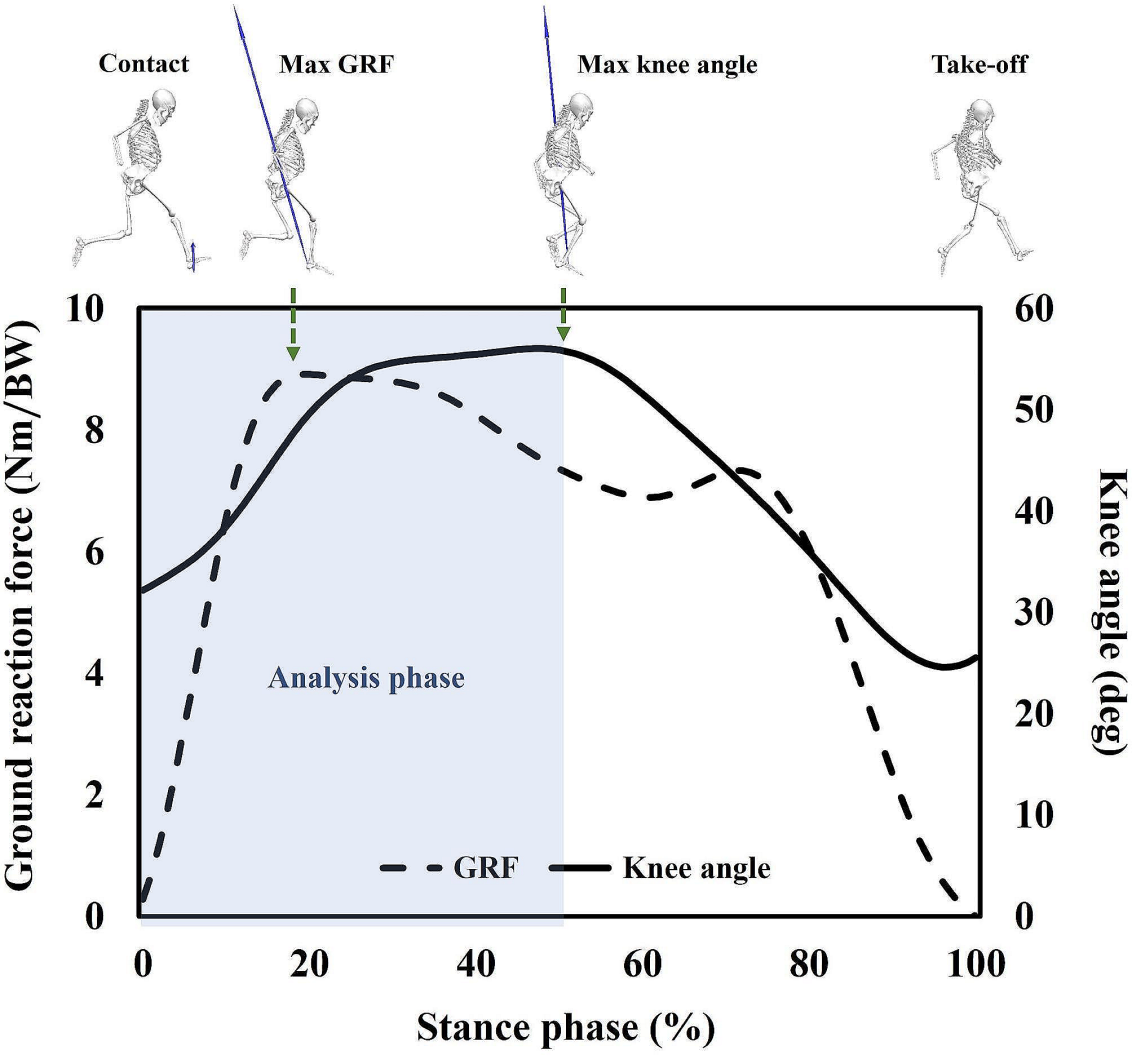
Analysis phase during cutting maneuver

The raw data of the acquired three-dimensional position and GRF were analyzed using the Visual 3D software (C-Motion Inc., Rockville, MD, USA) with a cutoff frequency of 15 Hz and a fourth-order Butterworth low-pass filter [33, 40]. In the present study, the analysis phase was defined as the area between the landing on the force plate and the maximal flexion of the knee joint with passive forces applied to the participant’s body (Fig. 3) [41]. Previous studies have reported an increased likelihood of knee joint injury in this area due to the entire body’s deceleration and the trunk’s reorientation in the target direction [2, 42].

Kinematic data for the right ankle, knee, hip was acquired and converted from quaternion to Euler angles (rotation sequence: XYZ) to allow comparison with the 3D motion analysis system [43]. The ground reaction force (GRF) of three directions during foot contact was normalized to body mass (BW) [12]. The joint moments were calculated using an inverse dynamics approach through Visual3d software and normalized to height (ht) and body mass [10, 36]. All variables were calculated as maximum (if +) or minimum (if -) from landing to maximum flexion of the knee joint [36].

Knee ligament and muscle modeling.

The OpenSim software (ver. 3.3, Stanford University, Stanford, CA, USA) was used to simulate the load on the knee joint ligaments in this study. This software models the respective muscles and ligaments in the body to facilitate the measurements of various parameters via the input of kinematic and kinetic data [44, 45]. This study used the GAIT2392 musculoskeletal model with 12 segments, 92 muscles, and 23 degrees of freedom [46]. Ten separate bundles were used to model the geometrical and mechanical properties of the ACL, PCL, MCL and LCL. The ACL consisted of an anterior bundle (aACL) and posterior bundle (pACL); The PCL consisted of an anterior bundle (aPCL) and posterior bundle (pPCL); the MCL was divided into a surface layer (iMCL) comprising aMCL, pMCL, and a posterior bundle, and a deep layer comprising an anterior (aDMCL) bundle and posterior (pDMCL) bundle, the LCL consisted of a lateral bundle [47]. All of the ligaments were calculated by summing all the individual bundles to calculate the integrated force at the corresponding ligaments. The data of ligament load at the knee joint were extracted through a process of body scaling, inverse kinematics (IK), inverse dynamics (ID), residual reduction algorithm (RRA), computed muscle control (CMC), and forward dynamics (FD) [36, 48].

The Hill model was used to calculate the ACL, PCL, MCL and LCL forces using the following equation [49].

$${f}_{m}^{*}=\left[{a}_{m}^{*}{f}_{lv}\left({l}_{m}^{*},{i}_{m}^{*}\right)+{f}_{psv}\left({l}_{m}^{*}\right)\right]\text{c}\text{o}\text{s}\left({a}_{m}^{*}\right)$$

$${f}_{m}^{*}$$= ligament force

$${a}_{m}^{*}$$= muscle activation

${f}_{lv}$= the dynamic force effected by the force-length-velocity curve of the Hill model

${l}_{m}^{*}$= the muscle length

${i}_{m}^{*}$= tendon velocity acting in the muscle direction

$${f}_{psv}$$= passive force

${a}_{m}^{*}$= the muscle pennation angle

First, the data of the participant’s body and the coordinates of the cutting maneuver were applied, and the extracted angles and angular velocities were used to calculate the force and moment. Subsequently, through residual reduction to minimize the error, the data for the final model, including individual muscle and tendon lengths and active and passive force, were extracted [48, 50].

### Statistical analysis

The pre-and post-test results of video feedback on preventing lower extremity injuries in cutting maneuvers were compared for statistical analysis. A paired t-test was performed to test the differences in dependent variables. The significance level was set at α = 0.05, and SPSS version 26.0 (IBM, Chicago, IL, USA) was used to perform all statistical tests. *Cohen’s dz* was used to evaluate the effect size, and one-dimensional statistical parametric mapping (SPM1D) was used to perform the paired t-test in time series analysis. All data were standardized to 100% of the time [51–53], whereas time points were validated according to the time series order using MATLAB 2023 (Mathworks, Natick, MA, USA).

## Results

### Kinematic variables: angle and angular velocity of lower extremities

The changes in kinematic variables of the lower extremity joints from video feedback were analyzed (Table 2). For the hip joint, the angles increased at flexion (t = 2.325, *p <* 0.05), abduction (t = − 4.251, *p <* 0.001), and external rotation (t = 4.009, *p <* 0.05), whereas the angular velocity increased at extension (t = 3.114, *p <* 0.05) after video feedback. For the knee joint, the angle decreased at internal rotation (t = 3.182, *p <* 0.05), the angular velocity increased at extension (t = − 2.943, *p <* 0.05). The ankle joint angle increased at dorsiflexion (t = − 2.516, *p <* 0.05), whereas the angular velocity decreased at dorsiflexion (t = 3.532, *p <* 0.05) and increased at eversion (t = 3.650, *p <* 0.05).

**Table 2:** Results of angle and angular velocity during cutting maneuver according to video feedback

		Pre-test	Post-test	t	*p*	Cohen’s *dz*
Hip						
Angle (deg)	Flexion	8.485 ± 6.128	11.464 ± 6.608	2.325	0.031*	0.661
	Abduction	0.275 ± 5.898	3.803 ± 5.380	–4.251	0.000*	0.854
	External rotation	–3.267 ± 5.740	–7.213 ± 4.196	4.009	0.001*	0.840
Angular velocity (deg/s)	Flexion	55.600 ± 107.873	110.74 ± 51.384	3.114	0.006*	0.823
	Abduction	–69.796 ± 132.818	–27.761 ± 91.441	1.508	0.148	0.521
	External rotation	–137.584 ± 118.930	–126.720 ± 110.138	–0.556	0.584	0.134
Knee						
Angle (deg)	Flexion	–31.449 ± 6.789	–29.721 ± 6.033	–1.291	0.212	0.381
	Adduction	6.603 ± 4.082	8.863 ± 3.727	–0.282	0.781	0.218
	Internal rotation	12.410 ± 7.739	9.682 ± 5.923	3.182	0.005*	0.803
Angular velocity (deg/s)	Extension	91.786 ± 177.328	185.45 ± 123.474	–2.943	0.008*	0.867
	Adduction	57.327 ± 116.043	9.703 ± 73.161	1.754	0.096	0.694
	Internal rotation	53.050 ± 174.754	1.106 ± 152.965	1.617	0.122	0.447
Ankle						
Angle (deg)	Dorsiflexion	12.050 ± 13.464	16.382 ± 13.474	–2.516	0.021*	0.755
	Eversion	–4.408 ± 5.171	–5.371 ± 6.747	–0.784	0.443	0.227
	Internal rotation	6.765 ± 5.181	4.947 ± 5.023	–1.893	0.074	0.504
Angular velocity (deg/s)	Dorsiflexion	425.318 ± 149.980	310.922 ± 209.397	3.532	0.002*	0.888
	Eversion	–156.907 ± 156.659	–288.826 ± 160.537	3.650	0.002*	0.876
	Internal rotation	8.913 ± 198.921	26.175 ± 177.262	0.314	0.757	0.130

*p < 0.05

**Fig. 4:**
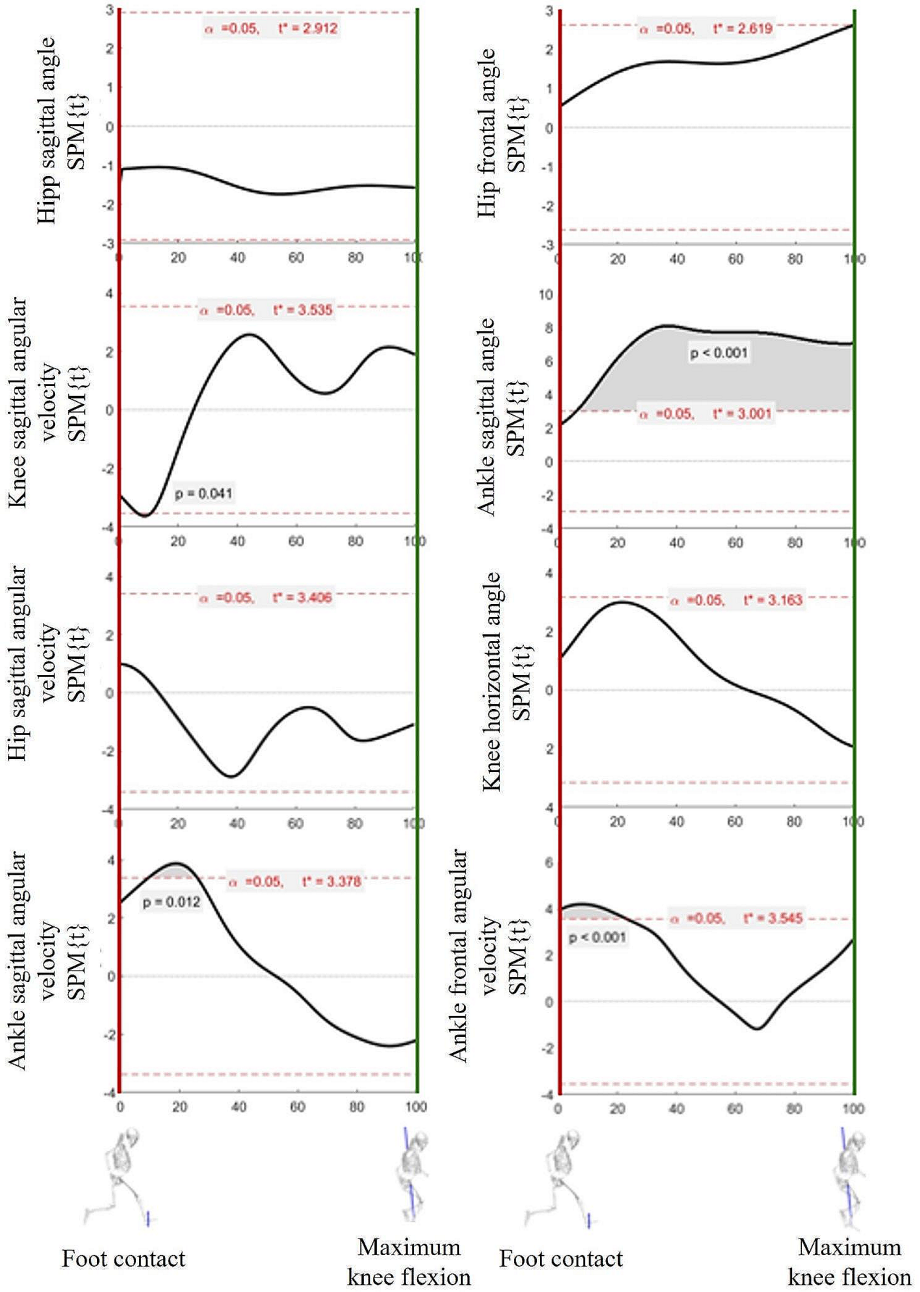
Mean differences of angle and angular velocity between the pre- and post-tests. When the SPM is greater than t*, a statistically significant difference is observed between the pre- and post-tests

Analyzing the temporal changes in kinematic variables according to video feedback showed statistically significant differences in the ankle joint angle and angular velocity on the sagittal plane and the angular velocity on the frontal plane (*p <* 0.05). The sagittal angle of the ankle joint differed at 6‒100% support (*p <* 0.001), whereas the angular velocity showed a statistical difference at 9‒26% support (*p <* 0.05). The frontal angular velocity showed a difference at 0‒23% support (*p <* 0.001) (Fig. 4).

### Kinetic variables: moment and ground reaction force

The changes in the GRF and lower extremity joint moments from video feedback were analyzed (Table 3). Statistically significant differences were observed in all three directions of GRF (AP: *p <* 0.001, ML: *p <* 0.05, V: *p <* 0.001). For the hip joint, the moment decreased at extension (t = − 2.335, *p <* 0.05) and external rotation (t = − 4.915, *p <* 0.001), but increased at adduction (t = − 2.215, *p <* 0.05). The knee joint moment decreased at extension (t = 2.941, *p <* 0.05), adduction (t = 5.042, *p <* 0.001), and external rotation (t = − 5.842, *p <* 0.001). The ankle joint moment decreased at abduction (t = 5.159, *p <* 0.001).

**Table 3:** Results of the moment and GRF during the cutting maneuver according to video feedback

	Pre-test	Post-test	t	*p*	Cohen’s *dz*
GRF (N/kg)					
Anterior-posterior	–4.538 ± 1.254	–5.851 ± 1.376	4.686	0.000*	0.953
Medial-lateral	10.856 ± 2.111	12.770 ± 2.194	–2.895	0.009*	0.830
Vertical	10.518 ± 1.462	9.221 ± 1.334	11.171	0.000*	0.927
Hip moment (Nm/[BW$\times$ht])					
Extension	–1.627 ± 0.644	–1.319 ± 0.285	–2.335	0.031*	0.619
Adduction	0.050 ± 0.076	0.128 ± 0.140	–2.235	0.038*	0.692
External rotation	–0.688 ± 0.259	–0.453 ± 0.251	–4.915	0.000*	0.921
Knee moment (Nm/[BW$\times$ht])					
Extension	0.715 ± 0.290	0.487 ± 0.236	2.941	0.008*	0.862
Abduction	–0.070 ± 0.080	–0.009 ± 0.052	5.042	0.000*	0.904
External rotation	0.254 ± 0.070	0.166 ± 0.125	–5.842	0.000*	0.869
Ankle moment (Nm/[BW$\times$ht])					
Dorsiflexion	–0.401 ± 0.190	–0.386 ± 0.171	–0.422	0.678	0.083
Inversion	0.568 ± 0.316	0.306 ± 0.246	5.159	0.000*	0.925
Internal rotation	0.038 ± 0.033	0.030 ± 0.064	0.583	0.567	0.157

ht: height; BW: body weight *p < 0.05

**Fig. 5:**
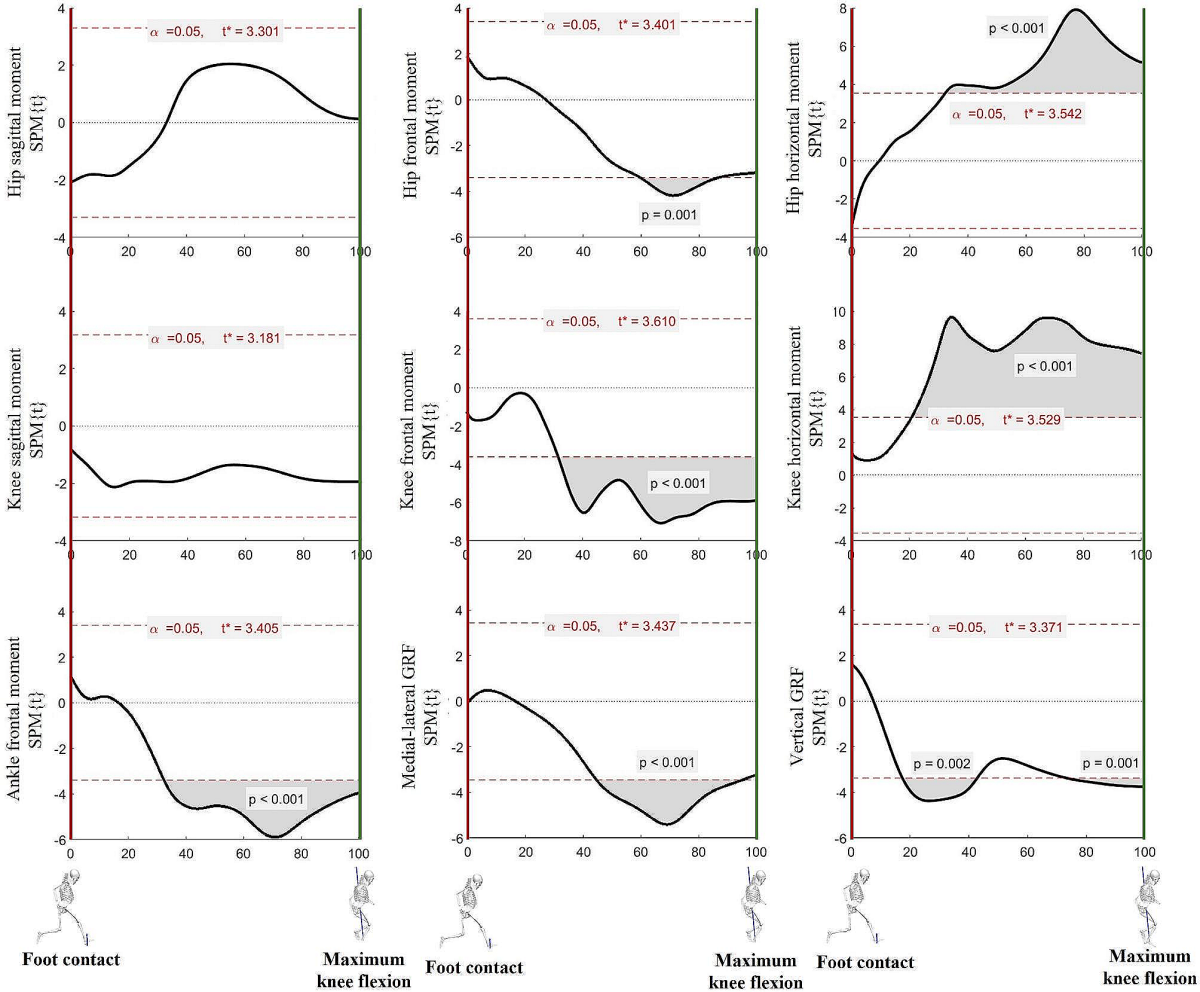
Mean differences of moment and GRF between the pre- and post-tests. When the SPM is greater than t*, a statistically significant difference is observed between the pre- and post-tests

Analyzing the temporal changes in the kinematic variables according to video feedback showed statistically significant differences in the hip frontal moment at 64‒87% support (*p <* 0.001) and hip horizontal moment at 32‒100% support (*p <* 0.001); in the knee frontal moment at 32‒100% support and knee horizontal moment at 21‒100% support (*p <* 0.001); and in the ankle frontal moment at 32‒100% support (*p <* 0.001). For the GRF, statistically significant differences were observed at 44‒95%, 17‒43%, and 73‒100% in the medial-lateral direction (*p <* 0.001), vertical direction (*p <* 0.05), and support (*p <* 0.001), respectively (Fig. 5).

### Ligament force

The changes in the forces generated at the knee joint ligaments according to video feedback were analyzed (Table 4). During the cutting maneuver task, the aMCL (t = 5.069, *p <* 0.001), iMCL (t = 3.800, *p <* 0.05), and pMCL (t = 2.427, *p <* 0.05) forces also decreased.

**Table 4:** Results of the ligament force during the cutting maneuver according to the video feedback

	Pre-test	Post-test	t	*p*	Cohen’s *dz*
aACL	1.386 ± 0.727	1.152 ± 0.587	1.150	0.265	0.354
pACL	0.349 ± 0.262	0.414 ± 0.215	–1.178	0.253	0.271
aPCL	0.171 ± 0.217	0.229 ± 0.385	− 0.888	0.386	0.186
pPCL	1.057 ± 0.676	0.855 ± 0.657	1.411	0.174	0.303
aMCL	0.768 ± 0.511	0.331 ± 0.451	5.069	0.000*	0.907
iMCL	0.989 ± 0.909	0.470 ± 0.612	3.800	0.001*	0.670
pMCL	0.801 ± 0.702	0.359 ± 0.360	2.427	0.025*	0.792
aDMCL	0.242 ± 0.245	0.184 ± 0.221	1.748	0.097	0.249
pDMCL	0.564 ± 0.700	0.410 ± 0.658	1.115	0.279	0.227
LCL	0.981 ± 0.578	1.082 ± 0.353	− 0.723	0.478	0.211

*p < 0.05

**Fig. 6:**
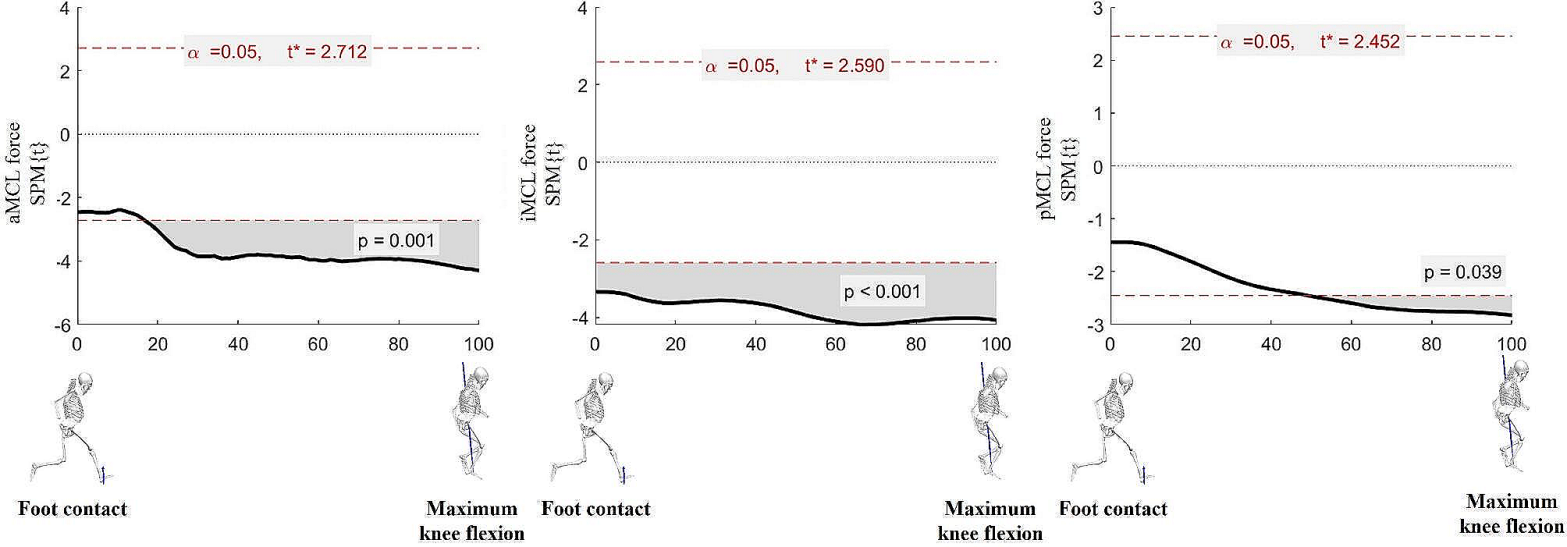
Mean differences of ligament force between the pre- and post-tests. When the SPM is greater than t*, a statistically significant difference is observed between the pre- and post-tests

Analyzing the temporal changes in the ACL and MCL forces according to the video feedback showed statistically significant differences at 17‒100%, 0‒100%, 50‒100%, and 0‒100% support for the aMCL (*p <* 0.05), iMCL (*p <* 0.001), pMCL (*p <* 0.05) force, respectively (Fig. 6).

## Discussion

The purpose of this study was to investigate the kinematic/kinetic variables of the lower extremity joints and load on the knee joint ligaments during the use of educational video program on cutting maneuvers. For this, a video demonstrating accurate cutting maneuvers was made with reference to previous studies, and the participants performed a cutting maneuver task five times before and after watching the video. The collected data were used to calculate the kinematic and kinetic variables of the lower extremity joints and to analyze the differences in the time series. The findings of this study verified to change kinematic and kinetic variables and decrease loads of knee ligaments by the short-term effects of video feedback.

During the cutting maneuver task, statistical differences were observed in all three planes (flexion, abduction, and external rotation) for the maximum angles of the hip joint. For the knee joint, the flexion angle did not increase, whereas the angular velocity showed increase. The increased hip flexion angle and knee flexion angular velocity suggest that the video feedback could induce changes in motor skills during the cutting maneuvers. The control of the hip joint in a cutting maneuver is particularly important, as the hip joint is subjected to a rapid accumulation of muscle fatigue, while affecting the trunk and knee joint movements, if interventions, such as feedback and training on cutting maneuvers, are not provided [54–56]. In a previous study comparing the hip flexion angle between the dominant and non-dominant leg, the hip flexion angle of the dominant leg was larger, which was attributed to the need to quickly shift the trunk in the forward direction while lowering the center of gravity [6]. The effect of the video program in the present study for the purpose of lowering the vertical position of the center of gravity was thought to be due to hip joint flexion. The increase in the flexion angle of the knee joint was attributed to ensuring a soft landing, but similar flexion angles were observed before and after watching the video program. This is likely due to the fact that the participants maintained the same speed during the cutting maneuver. However, the flexion angular velocity increased. In a previous study on female handball players, video feedback on jumping skills increased the angular velocity of the lower extremity joints during landing, and the subsequent jump height also increased as the impact on the body was absorbed [21]. Notably, the absorption is indicated by the increased angular velocity of the knee joint in the absence of a change in the knee joint angle. In this study, the increase in angular velocity was not large, which may imply the presence of the body’s shock absorption mechanism. If the angular velocity is mediated too rapidly, the extensor contraction of the quadriceps femoris muscle may increase with an increase in the anterior shear force of the tibia, resulting in a negative effect on knee joint ligaments [57]. After watching the video program, the dorsiflexion of the ankle joint increased and angular velocity decreased. In a previous study, the angle of dorsiflexion gradually decreased to 22.35°, 21.77°, and 16.04°, and the angular velocity decreased as the cutting angle increased to 0°, 30°, and 60°, respectively [58]. This decrease in the angular velocity upon dorsiflexion has been associated with an improvement in ankle stability during landing [19, 41]. In this study, the increased hip flexion and ankle flexion, increased angular velocity at the knee joint, and decreased angular velocity in dorsiflexion suggest that the impact on the body is dispersed through multiple joint movements, resulting in better-stabilized joint movements. Furthermore, hip abduction increased in the present study. When the load on the knee joint increases during the landing of a cutting maneuver, the hip abduction angle increases, whereas the knee joint angle increases concurrently in adduction and internal rotation [59]. However, in the present study, only the hip abduction angle increased, and the knee frontal and horizontal angles exhibited no increase. Notably, the decrease in the internal rotation angle of the knee joint has a significant effect in reducing the risk of non-contact type injuries [60]. When the foot is in contact with the ground and the tibia is fixed, minimal femoral rotation allows a stable landing and generates momentum to quickly run forward after a cutting maneuver [32]. Hence, due to the nature of the cutting movement, it is conjectured that the knee joint exhibited a relatively stable motion, while the hip abduction angle increased.

After watching the video program in this study, the vertical GRF decreased. Previous studies have demonstrated that the GRF increases after deceleration to enhance repeated momentum, which increases the risk of non-contact injury as the body weight increases the load after landing, thereby increasing the extensor contraction of the quadriceps femoris muscle and increasing the risk of noncontact injury, as the muscle is no longer capable of exerting a significant force [12, 61, 62]. An extensor contraction is a passive increase in the length of the muscle, which occurs in the opposite direction of the segmental movement and force [63]. Therefore, although fewer motor units are involved in muscle activity, a large tension is exerted to increase the likelihood of damage to the muscle microfibrils and limit their ability to support in the case of heavy load [63]. In this study, the vertical GRF decreased after the video feedback, suggesting that landing was performed through active contraction, rather than passive contraction. After the video feedback, the knee joint moments in extension, adduction, and external rotation decreased. Notably, the knee joint controls the trunk and pelvic rotations during cutting maneuvers to enhance the stability of the center of gravity, but with increased moments in internal and external rotations, the control becomes significantly difficult [56]. To reflect this in the video feedback, the foot orientation was adjusted to point inward, which resulted in a lower external rotation moment of the knee joint [36]. In a previous study, a cutting maneuver with the foot oriented in the forward direction led to a lower external rotation moment of the knee joint, consistent with the present study [36]. Notably, the increased moment of the knee joint in extension and external rotation could affect the risk of knee joint injury due to an increased length of the ACL [64, 65]. The video feedback in the present study likely led to a reduced the risk of injury through the organic movement and force of the lower extremity joints.

This study comprehensively analyzed the temporal changes in the ACL and MCL forces during cutting maneuvers using video feedback. The aMCL, iMCL, and pMCL forces decreased after watching the video program, and in a time series analysis, the differences were observed at 20‒30% for the vertical GRF, hip horizontal moment, and knee frontal and horizontal moments. In a previous study, the differences in the external rotation moment of the knee joint began to appear at 33% during cutting maneuvers, which is consistent with the present study [56]. These results suggest that notable differences in loading after a cutting maneuver likely occur at ~ 20%, closely resembling the time at which differences in the aMCL force on the anterior occur (17‒100%). If the horizontal moment is maintained in weight acceptance, the ACL and MCL are heavily loaded [56]. Additionally, at the time of highest loading at 70%, the hip frontal moment is critical in supporting the body weight, and in this study, a difference was observed at a similar point (64%) [66, 67]. Notably, when the hip frontal moment increases, the lower back muscles are used to minimize the hip abduction angle to prevent side-to-side movement [68]. The subsequent weight load increases gradually and a difference arises in the pMCL on the posterior, suggesting that the video feedback has an effect on reducing the load on the MCL.

The limitations of this study are as follows. First, the cutting maneuvers were performed in a laboratory; so they differ from those in real events. Second, our study is the exclusive inclusion of male participants. This gender-specific sample may restrict the generalizability of our findings. Third, the simulation was focused on noncontact movements, as it is not feasible to implement dynamic contact movements in controlled experimental conditions. Lastly, the participants in this study did not have a history of knee injuries, so the results may differ from those in individuals with knee joint ligament or cartilage injuries. Therefore, the results of this study should be used to develop prevention protocols and training programs for athletes without a history of knee ligament injuries.

## Conclusion

The purpose of this study was to identify how technical changes in cutting maneuvers induced by video program affect the kinematic and kinetic variables of the lower extremity joints and the load on the knee ligaments, and the following conclusions were drawn.

During a cutting maneuver, the hip joint angle increased in all conditions, the knee joint angle decreased at medial rotation with increased angular velocity. For the ankle joint at dorsiflexion, the angle increased, and the angular velocity decreased. Among the kinematic variables, the hip joint moment decreased at extension and external rotation and increased in adduction, whereas the knee joint moment decreased at extension, adduction, and external rotation. After watching the video program, the vertical GRF decreased, and the knee ligament force varied across the ACL, aMCL, iMCL, pMCL, and MCL. Thus, the video feedback on cutting maneuvers reduced the load on the lower extremity joints, which in turn reduced the load in the knee ligament.

The findings of this study verified the short-term effects of video feedback in preventing injuries. However, the goal of inducing motor skills changes is to ensure long-term effects through repeated learning. Thus, it is necessary to provide a new program for the prevention of lower extremity joint injuries with video feedback and, furthermore, with ‘eXtended Reality’ in accordance with the latest trends, from a long-term perspective.

## Data Availability

The datasets used and/or analysed during the current study available from the corresponding author on reasonable request.

## Abbreviations

GRF: Ground reaction force
ACL: Anterior cruciate ligament
aACL: Anterior ACL
pACL: Posterior ACL
PCL: Posterior cruciate ligament
aPCL: Anterior PCL
pPCL: Posterior PCL
MCL: Medial collateral ligament
aMCL: Anterior MCL
pMCL: Posterior MCL
iMCL: Surface layer MCL
aDMCL: Anterior deep layer MCL
pDMCL: Posterior deep layer MCL
LCL: Lateral collateral ligament.
SPM: Statistical parametric mapping
